# A method of decision analysis quantifying the effects of age and comorbidities on the probability of deriving significant benefit from medical treatments

**DOI:** 10.15256/joc.2017.7.93

**Published:** 2017-05-10

**Authors:** Stephen P. Fitzgerald, Nigel G. Bean, Ravi P. Ruberu

**Affiliations:** ^1^Department of General Medicine, The Royal Adelaide Hospital, Adelaide, South Australia, Australia; ^2^The School of Medicine, The University of Adelaide, Adelaide, South Australia, Australia; ^3^School of Mathematical Sciences, The University of Adelaide, Adelaide, South Australia, Australia; ^4^ARC Centre of Excellence for Mathematical and Statistical Frontiers, The University of Adelaide, Adelaide, South Australia, Australia; ^5^Division of Medical Subspecialties, Lyell McEwin Hospital, Elizabeth Vale, South Australia, Australia

**Keywords:** Aging, comorbidity, probability, quality-adjusted life years, treatment outcome, external validity

## Abstract

**Background:**

The external validity, or generalizability, of trials and guidelines has been considered poor in the context of multiple morbidity. How multiple morbidity might affect the magnitude of benefit of a given treatment, and thereby external validity, has had little study.

**Objective:**

To provide a method of decision analysis to quantify the effects of age and comorbidity on the probability of deriving a given magnitude of treatment benefit.

**Design:**

We developed a method to calculate probabilistically the effect of all of a patient’s comorbidities on their underlying utility, or well-being, at a future time point. From this, we derived a distribution of possible magnitudes of treatment benefit at that future time point. We then expressed this distribution as the probability of deriving at least a given magnitude of treatment benefit. To demonstrate the applicability of this method of decision analysis, we applied it to the treatment of hypercholesterolaemia in a geriatric population of 50 individuals. We highlighted the results of four of these individuals.

**Results:**

This method of analysis provided individualized quantifications of the effect of age and comorbidity on the probability of treatment benefit. The average probability of deriving a benefit, of at least 50% of the magnitude of benefit available to an individual without comorbidity, was only 0.8%.

**Conclusion:**

The effects of age and comorbidity on the probability of deriving significant treatment benefits can be quantified for any individual. Even without consideration of other factors affecting external validity, these effects may be sufficient to guide decision-making.

## Introduction

In making treatment decisions, doctors and patients must take into account relevant randomized clinical trials (RCTs) and systematic reviews. Relevance depends on external validity, or generalizability (i.e. whether the results can be applied to a definable group of patients in a particular setting in clinical practice and to individual patients) [1]. For example, how should a 75-year-old man with lung cancer, diabetes, hypertension, and atrial fibrillation (AF) be advised as to the benefits of treating hypercholesterolaemia, in his case?

There is concern amongst clinicians that external validity is often poor [1], particularly in the situation of multiple comorbidity [2–5]. Issues that may potentially affect external validity include the setting of the trial, characteristics of randomized patients, differences between the trial protocol and routine practice, outcome measures and follow-up, and adverse effects of treatment [1]. Another issue affecting external validity, one that has received little attention, is how qualitative aspects of treatment outcomes vary in different populations.

The fact that, in the context of comorbidity, the outcome of intercurrent death abolishes any treatment benefit has been recognized [6]. Decision analyses have indicated that treatments should be more conservative when longevity is limited [7, 8]. However, intercurrent non-fatal comorbidity also has an effect; it reduces, rather than necessarily abolishes, the magnitude of any treatment benefit. 

For an example of the effect of non-fatal comorbidity, imagine a treatment that prevents an illness resulting in breathlessness, which limits mobility to 50 metres. The treatment would provide enormous benefit to an athletic individual. If, on the other hand, an individual is limited to a mobility of 75 m by a comorbidity, such as arthritis, the treatment would still be beneficial, but less so. If a patient’s mobility is already restricted to 50 m or less, the magnitude of treatment benefit disappears. 

Another example of treatment benefit is the prevention of seizures allowing an individual to maintain their driving licence. This benefit is not available to an individual already unable to drive, on account of comorbidity. The prevention of seizures still has a benefit to that individual, but its magnitude is reduced by a quantum.

Although all patients are at risk of intercurrent death and impairment from comorbidity, this is particularly true for patients with known impairments and comorbidities [9–12]. This has been shown for both elderly (mean age 79 years) [9] and middle-aged (≥45 years) [10] patients. In practice, patients with comorbidities are commonly seen [5]. The probability of treatment benefit for these patients depends not only on the treatment-specific probability but also on the probability of remaining sufficiently well, despite their comorbidities, to be in a position to derive a significant magnitude of treatment benefit.

We have previously shown mathematically that comorbidities limit the benefits of treatments [13]. Previous studies of comorbidity, using quality-adjusted life years or the like as a metric of benefit, also indicate a reduction in treatment benefit. Cost–benefit studies have shown significant increases in this ratio in the context of comorbidity [14–16].

In this paper, we demonstrate that knowledge of a patient’s age and comorbidities allows an estimation of their probability of being in a given state of ‘wellness’ at a future date. From this estimation, we show that the probability of deriving given magnitudes of treatment benefit can be estimated. We provide a method to perform these calculations and demonstrate the principles of the method using the demonstration case in our introduction. We provide, in greater detail, examples of the application of this method to individual patients and to a population. These examples demonstrate how, in practical terms, the method might be applied, and how it might generate meaningful information. We propose simple variations of the method that might readily be applied in practice.

Information regarding benefit in terms of probability is important, as it is the way in which data are presented in trial reports. Furthermore, in contrast to the purpose of cost–benefit studies, which is primarily economic, the expression of information in terms of an individual’s probability of treatment benefit is fundamental to that patient’s assessment of, and consent to, therapies [17, 18].

## Methods

This study was approved by the Ethics Committee of The Royal Adelaide Hospital.

Our analysis is essentially a decision tree analysis, whereby all of the potential outcomes of a treatment are calculated [19]. For example, disease *a* might have outcomes *a*1, *a*2,* a*3, etc. Each of these outcomes has its own probability and utility state (a quantification of ‘wellness’ lying between 0 [death] and 1 [complete well-being], as determined *a priori*) [19]. Traditionally, the mean utility under different treatments is calculated and thereby the relative effects of the treatments are determined, i.e. the magnitude of the difference in treatment benefit is equivalent to the mean utility difference. 

We extended the traditional analyses by individually factoring in the potential outcomes of other comorbidities. Rather than the number of outcomes being limited to the number of outcomes associated with the treatment in question, we generated all of the combinations of possible outcomes taking other comorbidities into consideration. Thus, for comorbidity *b,* there may be outcomes *b*1 and* b*2, and for comorbidity *c*, there may be outcomes *c*1 and *c*2. Thereby, the number of potential outcomes for a patient with disease *a* and also comorbidities *b* and c, increases to include all of the possible combinations, e.g. *a*1,*b*1,*c*1, *a*1*b*1*c*2, etc. Just as in a traditional analysis, each outcome has its own *a priori* determined, or calculated, probability and utility states. 

In this paper, we only studied the benefits of treatments, and so our analysis excluded adverse outcomes (side effects, complications), due to a treatment. Thus, if *a*3 above represented an adverse outcome from the treatment of disease *a*, it would appear in a traditional decision analysis, but not in our analysis. Because we studied the outcome at a given time point rather than the accumulated benefit, we did not need to consider the order in which various outcomes might occur.

Rather than determining an average value, we chose to compare the distribution of benefit in the comorbid state with the distribution of benefit in the absence of comorbidity. The comorbid states vary in utility from 0% to 100%. We expressed the data in terms of the probability of utility being at least a given value. Though this calculation (the complementary cumulative distribution function of the quality of outcome) is cumbersome, it is mathematically simple and readily performed with a computer programme. Thus, for any patient, it becomes possible to state the probability of benefit for any given value. We appreciated that the minimum level of benefit that is considered ‘significant’ or ‘worthwhile’ is arbitrary and could, for example, be determined by consensus of professional bodies for guidelines, and/or by individuals for their own cases. This level could vary between [20] and within individuals for different conditions. We considered that, rather than diminishing the validity of this calculation, the validity is enhanced by this incorporation of personal values.

This method requires no additional assumptions to those of traditional decision analyses. The same probabilities and utility states used in other decision analyses/applications can be used as inputs. In fact, any idiosyncratic values may be chosen as inputs for utility states and for the probabilities of outcomes – the results would be valid for that hypothetical circumstance. The validity of the method (as for any decision analysis tool) is independent of the validity of the inputs. For there to be inter-observer agreement as to the validity of calculated results for particular individuals, however, there must be, as in all decision analyses, agreement with regard to the validity of the inputs.

### Demonstration of method

A 75-year-old male patient with lung cancer, diabetes, hypertension, and AF would like to better understand the benefit of starting statin therapy for hypercholesterolaemia. His healthcare professional has explained that it should lower his risk of having a myocardial infarction (MI). Evidence from large RCTs demonstrates that reducing cholesterol levels with a statin might decrease the 5-year risk of an MI by one-third [21–24]. For this male, a cardiovascular (CV) risk calculator [25] indicates that the 5-year risk of MI is high at 15%, and so, before considering any complicating factors, such as comorbidity, statin therapy in this case offers a 5% probability of benefit. Additionally, the average impairment score of a non-fatal MI has been reported to be 25% [26]. Therefore, before considering other factors and ignoring the possibility of fatal MI, the magnitude of treatment benefit is 25%.

However, as the patient is on treatment for lung cancer, and the 5-year outcome for survival is 50% [27], the 5% probability of benefit is subdivided such that there is a 2.5% probability of deriving benefit from statin treatment and of being alive (magnitude of benefit 25%), and a 2.5% probability of benefit, but also of being dead from cancer (magnitude of benefit reduced to 0%). In the absence of cancer treatment, the probability of death might be 60%, such that there is only a 2% probability of the 25% benefit.

If the patient is alive despite the cancer, there is also the consideration of the impairment of the cancer and its treatment. A usual method of assessing the magnitude of treatment benefit in the context of impairment from other conditions is to reduce the benefit in proportion [26, 28, 29]. If, therefore, the average impairment of the cancer and its treatment at 5 years is estimated at 40% [26], the magnitude of treatment benefit is reduced by 40% (the benefit of MI prevention is now 25% of 60%, i.e. 15%, rather than 25% of 100%). This reflects the fact that the patient is not in a position to derive the full benefit of MI prevention and, for example, may not derive the full benefit of freedom from fatigue and/or breathlessness. There is, thus, a partial retention of treatment benefit compared with the above-mentioned extreme situation of intercurrent death. The 2.5% probability of benefit from statin treatment, whilst being alive with cancer, now has a magnitude of only 15%, this accompanying the 2.5% probability of 0% benefit, derived with intercurrent death from the cancer.

The patient also has AF, and treatment (aspirin) and risk scoring (CHADS_2_ score 3) indicate that over 5 years there is a 20% probability of stroke [30–33], resulting in an average 50% impairment [26]. Consideration of this comorbidity results in the 2.5% probability of statin treatment benefit, whilst being alive with cancer treatment, being further subdivided. There is now a new category of benefit, the prevention of MI in an individual alive with both cancer and a stroke. The probability of this outcome is 0.5% (20% of 2.5%) and the relevant magnitude of benefit is 25% (the benefit of MI prevention) of the utility state of an individual with cancer and stroke (50% of 60%). This calculation results in a treatment benefit magnitude of 7.5%. Thus, the original 5% probability of benefit magnitude of 25% – the probability and magnitude of benefit of treatment in the absence of comorbidities – has been subdivided and reduced to the following:

- 2.5% probability of benefit + death from cancer (magnitude of benefit = 0%)
- 0.5% probability of benefit + alive with cancer + stroke (magnitude of benefit = 7.5%)
- 2.0% probability of benefit + alive with cancer without stroke (magnitude of benefit = 15%)

**Figure 1:**
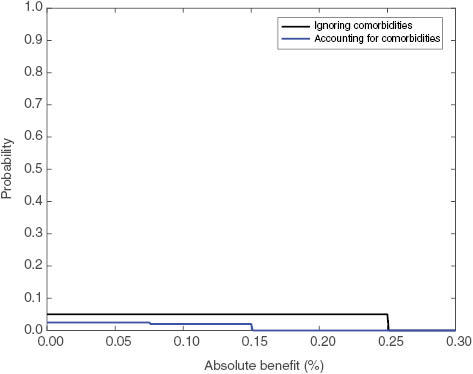
The probability of deriving any absolute level of benefit from statin therapy, with or without the consideration of comorbidities, for our demonstration case (75-year-old male with lung cancer, diabetes, hypertension and atrial fibrillation).

The patient can therefore be advised that his comorbidities have affected his probability of benefit from statin therapy, such that he has a 2.5% probability of deriving at least 7.5% benefit, a 2% probability of deriving at least 15% benefit, and a 0% probability of deriving greater than 15% benefit (and thus also 0% probability of deriving the full original 25% benefit magnitude). This can be expressed graphically (Figure 1). The patient can be advised that the 7.5–15% magnitude of benefit corresponds to the prevention of asymptomatic, or minimally symptomatic, ischaemic heart disease, in an individual without comorbidity [26].

Any of the patient’s other comorbidities and risks can be added to the calculation, providing there are estimates for the probability of the outcomes and their respective impairment values. Each new consideration progressively adds precision to, and lowers, the estimations of magnitudes of treatment benefit. Alternatively, the analysis may be simplified, such that only the major comorbidity, lung cancer, is considered. 

### Application of method

#### Patient selection

We obtained a subset of patient data from a larger project investigating the interactions between diseases, comorbidities, and treatments. For this larger project, we enrolled 50 consecutive patients seen in the Geriatric Clinic of the Royal Adelaide Hospital who satisfied the inclusion criterion of being on drug therapy for osteoporosis, hypertension, and hypercholesterolaemia, to serve as our study population. As this study is a decision analysis applied to a subset of individuals, the size of the parent sample is not of importance to this work. 

In this study, we highlighted four patients, deliberately chosen on account of their different comorbidity characteristics, to demonstrate the feasibility of applying the above method of comorbidity analysis to individual patients. There is no claim that these patients’ results can be generalized to other, dissimilar, individuals. We chose the patients to demonstrate the different ways in which comorbidity may affect treatment benefit, i.e. by death or impairment, and also to demonstrate the effect of an increased number of comorbidities. Any of the 50 patients could well have been highlighted, but the inter-individual differences might have been more subtle.

Table 1 summarizes the characteristics of the four selected patients. Patient 1 was 65 years old with initial impairment from diabetes (10%) and dementia (25%), and had an estimated 5-year dementia impairment of 60%. Patient 2 was 80 years old with minimal initial impairment from a previous fracture (3%) and mild chronic lung disease (10%). Patient 3 had even less initial impairment (3% from fracture) but, at 89 years of age, had a higher risk of death. Patient 4 was 83 years old with significant initial impairment from a lumbar plexopathy (40%) and cancer (15%), and at risk of stroke from AF. All four patients also had the three conditions required for inclusion in the study sample, i.e. they were being treated for hypercholesterolaemia, osteoporosis, and hypertension. 

#### Statin efficacy and comorbidity data

After analysis of the 50 participants’ medical histories, we calculated (by reference to the literature [21–24]) their individual expected 5-year risk of fatal and non-fatal MI, with or without therapy for hypercholesterolaemia. This calculation, based on the premise of no comorbidity, resulted in a value comparable to that of our demonstration case, as described in the first paragraph of the introduction.

Analogous to the consideration of comorbidity in the demonstration case, we then factored into our analysis the range of possible outcomes due to the effects of age, which, for the purposes of this paper, was treated as a comorbidity; the hypertension and osteoporosis present as per the selection criteria, and other comorbidities including AF, dementia, obstructive lung disease, cancer, diabetes, blindness, deafness, arthritis and Parkinson’s disease. 

For age, we estimated the 5-year risk of death [34]; for AF and hypertension, we estimated the 5-year risk of stroke [30–33, 35–37]; for osteoporosis, we calculated the 5-year risk for hip fracture [38–42]; and for dementia [43], obstructive lung disease [44], cancer [27, 45], and heart failure [46–49], we estimated the 5-year risks of progression and death. The risk of incident dementia was also considered [50]. For pragmatic reasons, we took the conservative course of assuming no progression in impairment or risk of death for diabetes, blindness, deafness, arthritis, and Parkinson’s disease. Similarly, we did not consider the patients’ other comorbidities, apart from severe neurological deficits in two patients (lumbar plexopathy and spinal infarction).

We assumed that the rate of accumulation of risk, and treatment benefit [21], was constant, and extrapolated the results of studies to 5 years as necessary. We chose 5 years as our time frame as this is commonly used in trials and in the quantification of CV risk [51]. As far as possible, all probabilities were checked to be based on censored data (i.e. data from patients who died during the relevant studies were not considered), and/or from trials in which the death rate, and thereby any potential effect of censoring, was small.

For the treatment of hypercholesterolaemia and the comorbidities, we accorded efficacies as generously as the literature would allow, with no reduction on account of the age and comorbidities of our patients *per se.* We considered the efficacies of different statins and different antihypertensives to be equivalent, but we differentiated the efficacy of antiresorptives (bisphosphonates/hormonal preparations) from that of supplemental calcium and vitamin D (Table 2).

#### Magnitude of treatment benefit data

We referred to the Guides to the Evaluation of Permanent Impairment (‘the Guides’) [26] for the quantification of the impairment of MI in the absence of comorbidity. This value became the magnitude of treatment benefit in the absence of comorbidity (‘the baseline benefit’). To translate ‘impairment’ to ‘utility’, ‘utility’ may be considered 1 − ‘impairment’. The baseline treatment benefit was 100% for prevention of fatal MI and 25% for prevention of non-fatal MI. 

We used the same source for the quantification of the impairment due to comorbidities. When impairment due to any other condition was uncertain, we used the lower end of the range quoted by the Guides.

The Guides, as also recommended elsewhere [28, 29], use a multiplicative method of combining impairments. In this mathematical method of determining the weighting of multiple pathologies, the weighting of impairment is multiplied by pre-existing well-being (1 – impairment). For example, what is considered to be 40% impairment in a perfectly well person is reduced to 32% impairment in an individual already 25% impaired. This prevents a sum of weightings exceeding 100% (equivalent to death). This multiplicative method also determines the magnitude of treatment benefit. If, as above, a potential impairment is reduced from 40% to 32%, the magnitude of benefit of a treatment that prevents this disability is also reduced from 40% to 32%.

#### Combining outcomes

In the context of multiple comorbidities, there are multiple possible outcomes, each with its own probability. When considering all the combinations of possible outcomes, we took the parsimonious option of considering them to be mutually independent. 

We took care not to double count impairments and risks. For example, we subtracted any component attributable to dementia and heart disease from age-related mortality on account of our separate calculation of mortality due to dementia and heart disease. When an impairment could have possibly been due to more than one condition, we recorded it under one heading only, i.e. the one we considered most likely to be correct. Thus, for our patients, we constructed a table documenting all of their treatments and comorbidities with the corresponding initial impairments (at the time of enrolment), and the probabilities for impairments and death at 5 years (Supplementary Methods 1). An overall summary of our patient sample is provided in Table 3.

### Analysis

#### Distribution of utility (well-being)

We first calculated the ‘comorbidity effect’ as the probability of being sufficiently able (i.e. not disabled/dead on account of other conditions), to derive at least a specified proportion of the baseline treatment benefit. More mathematically, we calculated ‘the probability of having other impairment less than or equal to that which allows the patient to derive at least a specified proportion of the baseline treatment benefit’ (see Supplementary Methods 2). 

In order to determine the first probability, we considered each individual in terms of the chances of death/disability from other conditions, i.e. without reference to the condition addressed by that treatment, in this case treated hypercholesterolaemia. We considered all of the possible combinations of other impairments. We calculated the probability and impairment magnitude of each combination. We could then calculate the probability of having impairment less than or equal to a given proportion (the complementary cumulative distribution function of the quality of outcome). Thus, for each patient, we could create a graph of their chances, with the x-axis showing the magnitude of benefit from 0% to 100% of baseline, and the y-axis showing the probability of deriving at least that magnitude of benefit. 

#### Distribution of treatment benefit

To quantify treatment benefit, we multiplied the above first probability (the comorbidity effect) by each individual’s baseline efficacy of the studied treatment (i.e. the probability of benefit ignoring the effects of comorbidity). This is just a multiple of the first probability (the complementary cumulative distribution function), but each individual has a different multiplier as each individual has a different CV risk, and thereby different baseline efficacy of treatment.

We also interpreted these results in absolute terms, the x-axis in this presentation showing the various degrees of absolute benefit i.e. 0–100% impairment benefit in absolute terms. This corresponds to the expression of benefit in the demonstration case.

#### Sensitivity analyses

We subjected our results to sensitivity analyses, changing our data regarding the baseline probability or efficacy of treatment up or down by 50%, and by eliminating the effect of age-related mortality. Using our assumption of a constant rate of accumulation of hazards and benefit, we also calculated the effects of reducing the time frame of analysis from 5 years to 1, 2, 3, or 4 years.

**Figure 2:**
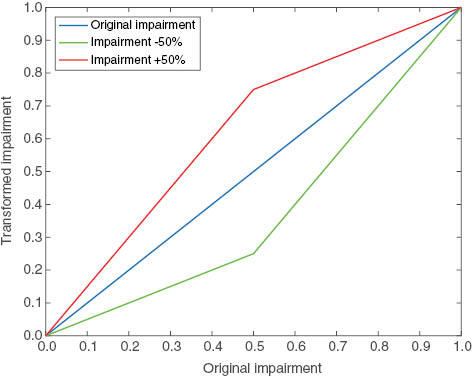
Transformation of impairment scores used in the sensitivity analysis.

In addition, we performed sensitivity analyses adjusting impairment scores and disease progression in a similar way to that for treatment efficacy (for disease progression we allowed for only a reduction). If the original impairment was less than 50%, we adjusted this up and down by 50%. However, for high impairment scores (over 50%), to avoid yielding an impairment score greater than 100% and to maintain symmetry with low impairment scores, we adjusted utility. Figure 2 is a graph of this transformation, indicating the sensitivity limits for each impairment value. As the impairment scores might be considered less objective than the other inputs, we also performed similar analyses whereby impairment values were reduced further, i.e. by 75% and 90%.

## Results

### Benefit of statin treatment in the absence of comorbidity

Our patients were at high risk of MI. The average untreated 5-year risk of MI was 14.8%, which was similar to that of the demonstration case. Thus, without considering comorbidity, the probability of deriving benefit from statin treatment was 4.9% (risk reduction by one-third). The magnitude of benefit was 25% for non-fatal MI and 100% for fatal MI. 

### Distribution of utility (well-being)

**Figure 3:**
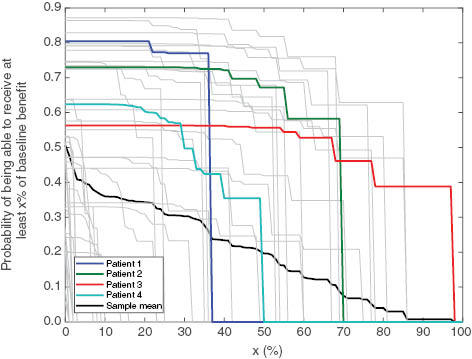
The probability for each of our 50 patients of being sufficiently well to be able to derive at least the specified proportion of the baseline impairment benefit for myocardial infarction prevention. The sample mean and our four selected patients’ curves are emphasized. Note the steep drops and long horizontal sections for the individual patients, which are caused by the assumption that any additional impairment is exactly the given average impairment, not a range of possible values from some unknown distribution. Were the literature to provide more detailed information as to this range in possible outcomes, then each such subset of the average outcome could be represented. The steep drops and long horizontal sections would then be broken into multiple smaller steps, such that the graphs would eventually become smooth decreasing curves.

The comorbidity effect (i.e. the probability of being sufficiently able [i.e. not disabled/dead on account of other conditions]), to derive at least a specified proportion of the baseline treatment benefit was different for each of the 50 patients. Figure 3 shows the probability distribution for each individual’s benefit of MI prevention with statin therapy. Also shown is the mean for this sample of 50 patients. The height of each individual intercept on the y-axis indicates the probability of being alive at 5 years (and thus the probability of deriving *any* benefit from treatment); the intercept on the x-axis indicates the maximum possible state of utility (1 – impairment) for that individual at 5 years. This latter value was largely a function of the initial state.

The profile of Patient 2 indicates that he was in a position to have the greatest probability, of the four selected patients, of deriving a benefit comparable with baseline (Figure 3). This profile reflects that he was minimally impaired and had a reasonably good 5-year prognosis. Though Patient 3 also had only mild baseline impairment, the probability of deriving any level of significant benefit was greatly reduced by the age-related risk of death. Patient 1, though having a reasonable longevity chance, was unable to derive benefit above 45% baseline benefit on account of baseline impairment. The profile of Patient 4 had more segments plotted. As each segment corresponds to a discrete utility state, such an increase in the number of segments indicates that Patient 4 had more known comorbidities, and therefore, more combinations of comorbidity outcomes, and thereby, a greater range of quantifiable potential states of impairment.

### Distribution of treatment benefit

**Figure 4:**
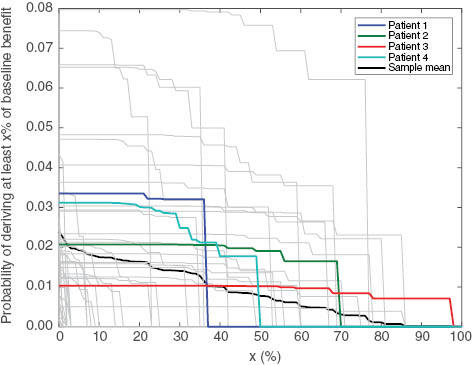
The probability of deriving a specified proportion of a baseline impairment benefit for each of our 50 patients. This probability is a subdivision of the baseline efficacy. The sample mean and our four selected patients’ curves are emphasized.

Figure 4 displays the probabilities of deriving at least a specified proportion of a baseline impairment benefit. These probabilities are low on account of both the modest baseline efficacies of treatment and the reduction by the effect of comorbidity. Because each patient has a different baseline efficacy of cholesterol-lowering therapy, on account of different CV risk, the curves are not identical to those of Figure 3.

Patient 4, for example, who, in the absence of comorbidity, would have had a 5% probability of benefit, only has a 3.1% probability of any benefit (where the curve of Patient 4 crosses the y-axis, this value having been reduced from 5% by the risk of death). Furthermore, there is only a 1.8% probability that the magnitude of benefit will exceed 50% of the baseline benefit. In the absence of comorbidity, the full 5% probability of benefit would be for a benefit magnitude of 100% baseline benefit. Of the four highlighted patients, Patient 2 had the greatest probability (1.9%) of deriving a magnitude of benefit of at least 50% baseline (as well as a similar probability of deriving a magnitude of benefit of at least 60–70% baseline).

**Figure 5:**
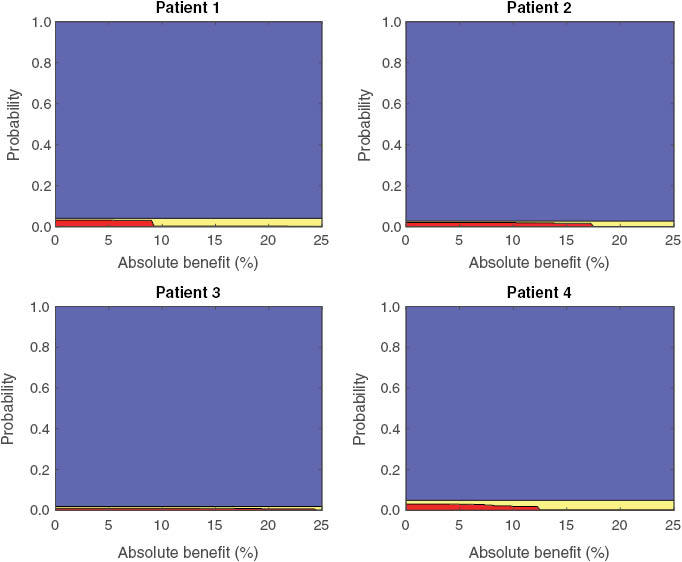
The probability of deriving any absolute level of benefit. Because the probability of any treatment benefit in any individual is at best around 15% (the efficacy of statin therapy), the most likely outcome of treatment is no benefit. The large blue section of each graph represents this probability. The remainder of each graph represents the probability of benefit from statin therapy; this is subdivided into two sections, indicating the probability that the benefit magnitude will (red), or will not (yellow), be significant. As one chooses a higher magnitude of benefit deemed significant, the probability of deriving such a benefit diminishes. The probability of benefit actually extends beyond a magnitude of 25%, but is too small to be observed on this scale.

Figure 5 shows the information in terms of *absolute* benefit values for the patients. Note that, as statin efficacy is, at best, less than 15%, the most likely outcome is no treatment benefit, and that the probability of any benefit is subdivided into the chance of benefit of ‘significant’ and ‘non-significant’ magnitude. Any increase in the minimum magnitude of benefit deemed significant (the x-axis) decreases the probability of deriving that benefit.

The red section of Figure 5 corresponds to Figure 1 from the demonstration case. For Patient 4, the probability of significant benefit becomes very small once a value of 12.5% is exceeded on the x-axis; i.e. the bulk of the modest baseline efficacy provides an absolute benefit magnitude between 0% and 12.5%. This range corresponds to the baseline impairment of asymptomatic, or minimally symptomatic, heart disease.

### Sensitivity analyses

**Figure 6:**
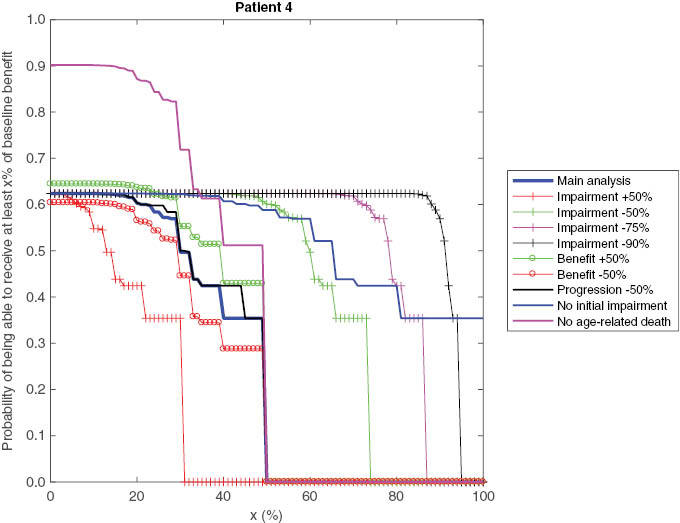
Sensitivity studies for Patient 4. Adjustments, up and down, were made to estimates of treatment efficacy and impairment scores. Age-related death, and initial impairment were removed from consideration, and disease progressions were reduced, these all being adjustments to mitigate the effects of coomrbidity. The main analysis refers to that shown in Figure 3.

Figure 6 shows that our conclusions were robust, requiring major changes to the input data to generate major changes to the probability distributions. The sensitivity studies reflected the different dependence of each patient’s outcomes on impairment estimations and treatment efficacy estimations. As the sensitivity studies result in complex graphs, the graph of only Patient 4 is shown to serve as an example.

**Figure 7:**
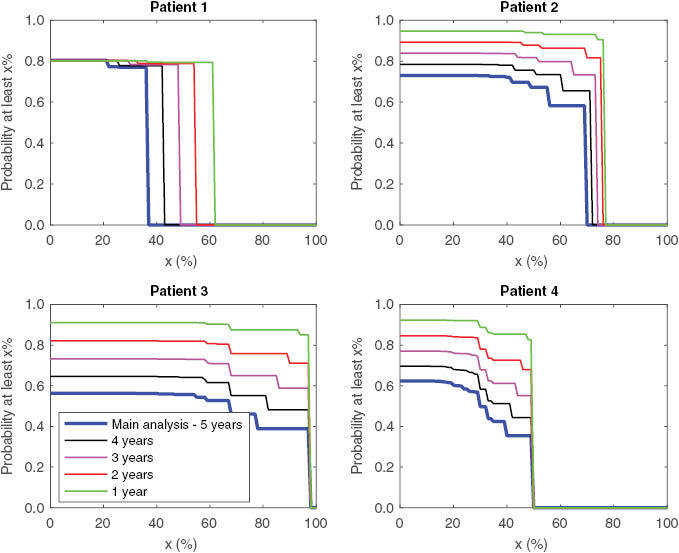
Sensitivity studies of the effect of changing the time span of analysis from 5 years to 1, 2, 3, and 4 years. The main analysis refers to that shown in Figure 3.

Figure 7 shows that when considering a longer time frame of analysis, the effect of comorbidity in diminishing the magnitude of treatment benefit increases. This is to be expected, as with the passage of time, there is more opportunity to accumulate impairment. For example, in an extreme scenario of a 20-year outlook, one would expect treatment benefit magnitude at that time point to be universally zero, as all our geriatric patients would be expected to have died. This was particularly true when the comorbidity effect was determined more by age-related risk of death rather than by baseline impairment. Although there is a decreased comorbidity effect with shorter time, the baseline treatment benefit decreases commensurately. The benefits of treatments tend to accumulate proportionately over time, and in particular this has been shown for statin therapy [21].

### Simpler expression of results

**Figure 8:**
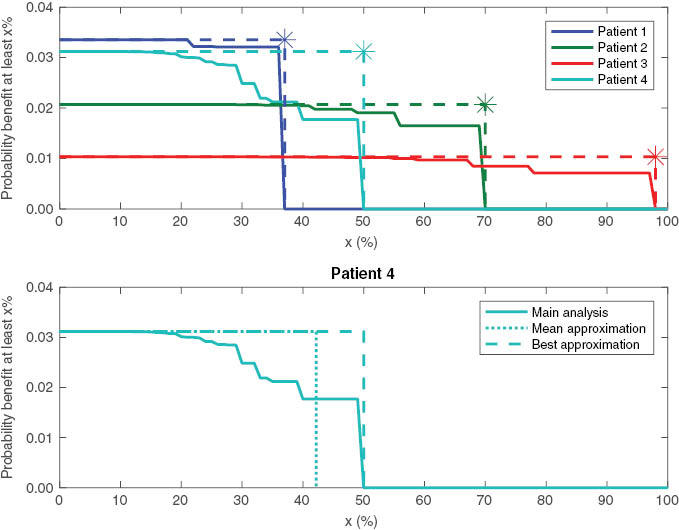
Expression of data as single point estimates. Estimates were calculated using a combination of the probability of being alive at 5 years and deriving any treatment benefit, with the *best* anticipated utility for our four selected patients (upper panel). These point estimates are at the intersection of the horizontal and vertical lines, and are represented by the asterisks. Patient 4, for example, could have his probabilities condensed to a point estimate of just over a 3% chance of a benefit magnitude of at least 50% of the baseline benefit (upper panel). For Patient 4, point estimates (asterisks not inserted) were also calculated using the *mean*, rather than the *best*, anticipated utility (lower panel). The just over 3% chance is now for a benefit magnitude of only at least 42% of the baseline benefit. The solid lines in each panel represent the main analyses of this paper. The main analysis refers to that shown in Figure 4.

Rather than having a multiplicity of possible chances of benefit, depending upon the magnitude of treatment benefit considered significant, the chances can be approximated to a single point estimate. One such estimate, applied to the chances demonstrated in Figure 4, results from the combination of the probability of being alive at 5 years and deriving any treatment benefit, with the* best* possible anticipated utility (Figure 8 upper panel). This latter variable can be further simplified to the utility state at time zero. This estimate results in a conservative measure of the effects of comorbidity.

This approximation is quite close for Patients 1, 2, and 3, as they have fewer comorbidities. The approximation is less accurate and more conservative for Patient 4. For this patient, an alternative point estimate results from the use of the *mean,* rather than the* best,* anticipated utility (Figure 8 lower panel).

Analogous point estimates may be obtained for the probability of absolute treatment benefit (corresponding to Figure 5).

### Full patient group results

For our full sample of 50 patients, there was, on average, only approximately a 20% probability of being able to derive at least 50% of the magnitude of the baseline benefit (Figure 3). After incorporating the baseline efficacy of treatment, the average probability of deriving at least 50% of a positive baseline impairment benefit was only 0.8% (Figure 4). In the absence of comorbidity there would have been on average a 4.9% probability of a benefit magnitude 100% of baseline. 

## Discussion

Using data from standard sources, and without making any new assumptions, we have presented a method by which the effect of comorbidity on the magnitude of treatment benefits can be quantified in terms of the change in the probability of benefit. Our examples show that there is interindividual variation in the effects of comorbidity, and that these effects can be significant, such that treatments that offer modest probability and/or modest magnitudes of benefit in the absence of considerations of comorbidity, may offer very low chances of significant benefit in the context of comorbidity. 

Our results were obtained by accepting trial results and guidelines at face value, indicating that, even in the absence of estimations of the effects of the other factors that limit the external validity of trials and guidelines, external validity can be shown to be poor, merely by considering the magnitude of treatment benefit. Furthermore, apart from just showing that external validity is poor, this consideration may provide sufficient information to advise individual patients, who are elderly and/or with multiple morbidity, as to the degree to which trials and guidelines might overestimate their individual probability of significant treatment benefit. The consideration of the other factors affecting external validity would complement the use of our method.

The merit of a medical therapy is first assessed on the basis of the associated probability of benefit in the context of its risks [52]. If the probability is considered attractive, its cost then determines whether or not the therapy can be justified economically. When considering comorbidity, our method allows the reassessment of the probability of treatment benefit, in terms analogous to the original studies, so that these can be confirmed to be attractive prior to the consideration of the revised cost–benefit. Any need to refuse/not offer treatments on the basis of cost–benefit considerations in the context of comorbidity is thereby likely to be diminished.

Our analysis of patients on statin therapy for hypercholesterolaemia has demonstrated that, if only cardiovascular risk (i.e. no comorbidity) had been considered, these patients might have been judged to have had a good probability of benefit, in accordance with guidelines [53]. However, when comorbidity was considered, statins offered, on average, only very low chances of significant benefit, similar to those for patients with much lower cardiac risk and no comorbidity. Such patients at lower cardiac risk, and with lower potential for benefit, would not normally be considered candidates for therapy [53]. 

Our patients’ anticipated benefit was assessed for a 5-year outlook, whereas guidelines suggest a 10-year outlook [53]. As our patients were older individuals with comorbidity, their probability of benefit over a subsequent 5-year period would be expected to be even lower, such that the 10-year benefit would be well below the minimum level of 10-year benefit thought to justify statin therapy [53]. Furthermore, even this minimum level is low; the minimum risk level is 7.5% over 10 years, such that the expected benefit would be approximately 2.5% over 10 years. Even in the best circumstances, the benefit of statins [54], like the benefits of other recommended treatments [55], are often not considered by patients [54] or the general public [55] to be sufficiently great to justify the promotion of such treatments.

We have thus shown that CV risk assessment alone may not provide the best measure for selecting patients for this therapy, but rather that such an assessment would be better done together with a quantitative assessment of the effect of comorbidity. Patients with a high cardiac risk and a low comorbidity burden stand to gain the most from statins. A patient with high cardiac risk may still be in a position to derive significant benefit, despite comorbidities. The demonstration patient, and perhaps Patient 4, might be such individuals, depending upon the level of benefit magnitude deemed significant. Patient 2, on account of a moderately high MI risk and a relatively low comorbidity burden, also maintained a modest level of treatment benefit. Additionally, other patients in our sample of 50 individuals demonstrated even better levels of treatment benefit (Figure 4).

The opportunity to incorporate patient-centred outcomes, engage patients in decision-making, and enhance physician–patient shared decision-making would be greater using such an assessment. This approach, like other decision analyses [56], cannot itself provide a definitive answer as to whether the probability of benefit justifies a given treatment. There may be guidelines, but individual practitioners and patients may prefer to set their own threshold of significant benefit and consider the probability themselves. 

Our method is an extension of the consideration of multiple competing outcomes. It has been pointed out that in a population at significant risk of death, the baseline risk of any outcome is likely to be overestimated if risk is extrapolated from studies using censored data [6]. In these circumstances, the probability of any therapeutic benefit is correspondingly reduced. We have considered how non-fatal comorbidity, an additive rather than a competitive entity, also restricts the benefit of any intervention. 

Ideally, all of a patient’s comorbidities would be included in an analysis using this method. For pragmatic reasons, we limited the number of comorbidities and outcomes that we analysed. This rendered our results conservative. Further limitation of the number of comorbidities considered, even down to a single major comorbidity, provides a means by which any practitioner might relatively easily apply the method without assistance of a mathematician or a computer programme. Our examples of simpler expressions of the results of a fuller calculation may also help with applying the method in practice. 

We have only studied the *benefits* of treatments in the context of comorbidity. We did not analyse costs and risks in the same way, but we know from previous studies [14–16, 57] showing a fall in cost-benefit, that, in the context of comorbidity, costs do not fall proportionately with benefit. Furthermore, even in the absence of consideration of risk, treatments require a threshold level of benefit to be judged worthwhile [54].

The source of our impairment percentages implies that all impairments of the same absolute value are equal, and that the same pathology causes different incremental impairments, depending upon other contemporaneous impairment. This multiplicative method is also endorsed elsewhere [28, 29]. We believe this relationship is rational, expressing mathematically the generalization that if one is unable to perform a function on account of impairment in one system, a similar dysfunction due to impairment in another system will be, to some extent, redundant. The use of different inputs would result in different estimates of the comorbidity effect, and different methods of calculating the impairment of combined conditions would necessitate different methods for generating the probability calculations. All resulting estimates of the comorbidity effect would be valid. Potentially, the impairment values for all combinations of conditions might be known empirically. 

If the impairments of comorbidities were to be regarded as combining strictly additively, the comorbidity effect would disappear and trial data and guidelines could be applied without consideration of comorbidity. There is, however, no inherent justification for such an assumption, the shortcomings of which have previously been described [29]. In particular, it is not possible for the outcome of death to be additive [29] (if more than one fatal outcome is averted only one life is saved).

We assumed that all outcomes of different conditions are mutually independent. In general, this is a parsimonious assumption, e.g. there are no data to indicate that a patient with osteoporosis has greater or lesser protection from an MI with statin therapy compared with a patient without osteoporosis. If there is evidence that comorbidities affect the outcomes of the treatment in question, then this can be incorporated into the initial individualized data, as we did in assessing MI and AF-related stroke risk, for example. Our impairment scores for multiple comorbidities were based on the impairment scores for each of the individual comorbidities. To the extent that the impairment resulting from individual comorbidities may, on account of clinical, rather than mathematical, interactions, be even more severe in the presence of other comorbidities, our results are conservative. For example, in our demonstration case, if the outcome from stroke was exacerbated by the presence of the lung cancer, then the utility state in these circumstances would be even worse than what we have allowed, and, the benefit from statins would be even less. Any lack of independence of the probability of adverse outcomes of different comorbidities would increase the probability of impairment at both ends of the spectrum. 

Our sensitivity analyses suggest that results obtained using our method are likely to be robust, i.e. likely to withstand variations in inputs. We believe that the ±50% sensitivity analyses that we performed for efficacy suffice, as these inputs are trial-based. Our sensitivity analyses suggest that the inputs from different sources for risk estimation and treatment effect would have to be large to change our results significantly. One could argue that our impairment estimates, though based on empirical data and taken from a recognized reference, are arbitrary and possibly inaccurate. However, our sensitivity analyses again indicate that a considerable overestimation of impairment scores is required to minimize the comorbidity effect. Such changes to our impairment scores are not only unrealistic, they would also cast doubt on the value of the medical interventions in the first place, i.e. the impairment they prevent would be small. Our estimates of the effects of age and comorbidity may be considered conservative, as there are additional impairments that might have been considered. These include any impairment due to age, *per se* (i.e. only the age-related death risk was included), and the effects of psychosocial and unknown future comorbidities.

In accordance with other methods analysing the effects of comorbidity [14–16], our method demonstrates that treatments to prevent an illness in an older person with comorbidity are likely to be less advantageous than the same treatments given to a younger, otherwise healthy, person. Our method faces similar limitations and challenges to those of more conventional economic studies. These include variation in health state utilities (which in our source would translate to impairment values), concerns of fairness of treatment, and the possible underestimation of the individual utility of health gains when treatment potentials are somewhat limited [58]. To some extent, our method, by providing a conservative measure of comorbidity, might allay these concerns.

## Conclusions

Many practitioners are intuitively aware of the effects of age and comorbidity on the probability of the benefit of medical treatments, and adjust patient care accordingly. The formal description in this paper of the relationships underlying these effects articulates the complexity of this practice and opens this aspect of ‘clinical judgement’ [59] to quantification and academic discussion. In particular, we have demonstrated that decision-making regarding the probability of the benefit of a medical intervention may involve, in addition to consideration of the risk of the specific disease in question and trial-based probabilities, consideration of initial impairment and age, and comorbidity-related death and impairment threats. These factors are amenable to quantitative analysis, rather than being restricted to qualitative description. Such an analysis complements cost–benefit, and other external validity, considerations. 

## Figures and Tables

**Table 1:** Characteristics of the four selected patients.

Patient	Age, years	Sex	Comorbidities
1	65	Male	Osteoporosis, hypertension, hypercholesterolaemia diabetes, dementia
2	80	Female	Osteoporosis, hypertension, hypercholesterolaemia, fracture, chronic obstructive pulmonary disease
3	89	Female	Osteoporosis, hypertension, hypercholesterolaemia, fracture
4	83	Male	Osteoporosis, hypertension, hypercholesterolaemia, lumbar plexopathy, cancer, atrial fibrillation

**Table 2:** Examples of treatment efficacies obtained from the literature.

Condition	Outcome risk	Treatment	Risk reduction, %	References
Hypercholesterolaemia	Myocardial infarction	Statin	50	[19–22]
Hypertension	Cerebrovascular accident	Antihypertensive	33	[26]
Osteoporosis	Hip fracture	Antiresorptive agent	50	[32, 35]
		Calcium ± vitamin D	20	[34, 35]
Heart failure	Death	b-blocker	33	[41]
		ACE inhibitor	33	[42]
		ACE inhibitor + b-blocker	50	[41, 42]
Atrial fibrillation	Cerebrovascular accident	Anticoagulation	60	[30]
		Antiplatelet	22	[30]

ACE, angiotensin-converting enzyme.

**Table 3:** Demographics of study population.

Demographic	
Mean age, years (range)	80.7 (65–92)
Sex, *n*	
Male	23
Female	27
Number of medications, mean (range)	10.4 (3–19)
Number of comorbidities, mean (range)*	7 (3–10)
Residential status, *n*	
Own home	42
Nursing home	8
Mean initial impairment, %	57.45
5-year conditional impairment, % (if alive)	66.73
5-year mortality, %	49.05
Median life expectancy, years	∼5
Charlson comorbidity index, mean (range)	3.34 (0–7)
Charlson age-adjusted comorbidity index, mean (range)	6.94 (4–11)

^*^All of the patients had at least the three comorbidities necessary for inclusion in the study, i.e. treated hypercholesterolemia, osteoporosis, and hypertension.
